# Stewart-Treves syndrome: A fatal complication of rheumatoid arthritis-associated massive lymphedema

**DOI:** 10.1016/j.jdcr.2025.12.023

**Published:** 2025-12-24

**Authors:** Shatila Torabi, Amin Saber Tanha, Naser Tayyebi Meibodi, Zahra Rafiei Vardanjani

**Affiliations:** a Department of Dermatology, Imam Reza Hospital, Mashhad University of Medical Sciences (MUMS), Mashhad, Iran; b Nuclear Medicine Research Center, Mashhad University of Medical Sciences (MUMS), Mashhad, Iran; c Department of Pathology, Faculty of Medicine, Mashhad University of Medical Sciences (MUMS), Mashhad, Iran

**Keywords:** Angiosarcoma, Oncology, Rehabilitative medicine, Secondary lymphedema, Soft tissue sarcoma

## Introduction

Stewart-Treves syndrome (STS) represents a rare and aggressive complication characterized by the development of cutaneous angiosarcoma in the context of chronic lymphedema, most commonly following mastectomy and axillary lymph node dissection for breast cancer.^1^ This entity, first described in 1948, underscores the malignant potential of prolonged lymphatic stasis, with angiosarcomas exhibiting rapid progression, frequent metastasis, and dismal prognosis despite multimodal therapy.^2^ This case report presents an unusual instance of epithelioid angiosarcoma (EAS) arising in rheumatoid arthritis (RA)-associated lymphedema of the lower extremity in a relatively young patient, emphasizing the need for vigilant monitoring and proactive management in such high-risk populations.

## Case presentation

**Fig 1:**
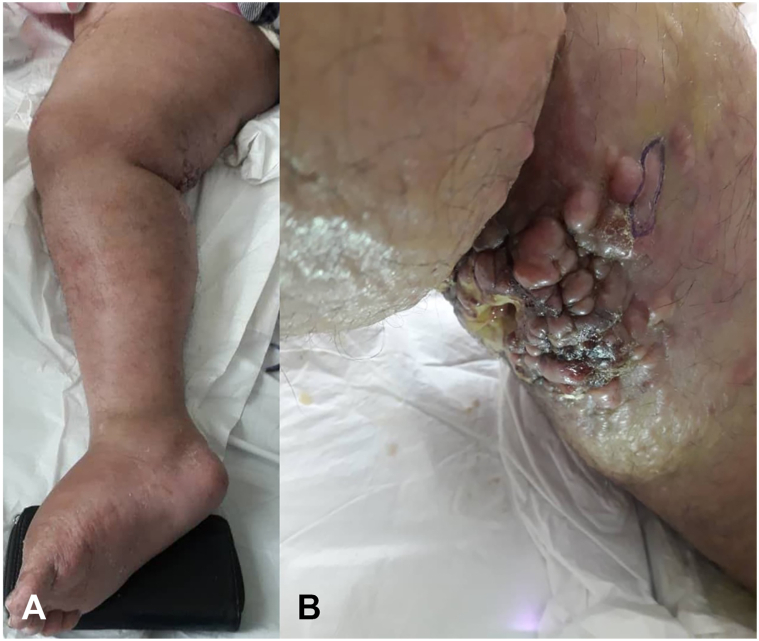
The right lower extremity demonstrates diffuse edema involving the entire limb **(A)**. Clinical examination reveals induration localized to the medial thigh and popliteal regions, with multiple firm, *skin-colored*, infiltrative nodules evident on visual inspection **(B)**.

**Fig 2:**
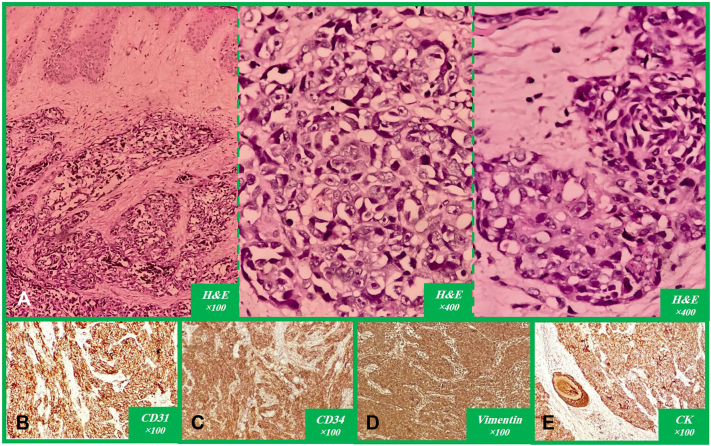
An invasive malignant tumor composed of solid and fascicular pattern and forming irregular vascular spaces lined by atypical cells, predominantly epithelioid and less frequently *spindle-shaped* within a fibromyxoid stroma. Some tumor cells contain intracytoplasmic vacuoles, and scattered mitotic figures and necrosis are present. There is also extension of the tumor cells into the epidermis with ulceration, as well as invasion into the hypodermis showing perineural infiltration **(A)**. On immunohistochemical staining, the tumor cells were positive for CD31 **(B),** CD34 **(C),** vimentin **(D),** and CK **(E),** while negative for S-100, Melan-A, SOX10, p63, EMA, and CEA. *CEA*, Carcinoembryonic antigen; *CK*, cytokeratin; *EMA*, epithelial membrane antigen.

A 40-year-old woman with an 8-year history of RA, managed with sulfasalazine and prednisolone, presented with firm, tender, erythematous nodules with purulent and bloody discharge in the popliteal region of her right lower limb, which was affected by progressively worsening edema. She had been relatively bedridden for the past 3 years due to painful hip joints affected by severe degenerative changes; however, she declined to undergo hip arthroplasty. Physical examination revealed induration in the medial thigh and popliteal region, accompanied by skin-colored, firm infiltrative nodules without any epidermal abnormalities, scars, erosions, or vesicles in most of them; although, some of the lesions were ulcerated and secondarily infected, with surrounding cellulitis and lymphangitic changes (Fig 1). A differential diagnosis for these lesions included infectious granulomas, cutaneous lymphoma, cutaneous metastases, and soft tissue sarcoma. To make a definite diagnosis, a deep incisional biopsy was taken from the infiltrative nodule in the indurated area located in the popliteal fossa. Histopathological examination of the specimen demonstrated epithelioid and spindle cells arranged in fascicular, trabecular, and nodular patterns with vascular spaces, extending from the ulcerated epidermis into the adipose tissue and hypodermis, accompanied by scattered mitoses and necrosis within a fibromyxoid stroma, moderate lymphoid and sparse plasmacytic infiltrates, extensive hyalinized collagen fibers, perineural invasion, and pseudo-epitheliomatous hyperplasia of the epidermal margins without dysplasia or melanocytic proliferation. Immunohistochemical analysis demonstrated negativity for P63, epithelial membrane antigen, carcinoembryonic antigen, and SOX10; weak to moderate positivity for S100 and Melan-A; and diffuse positivity for Vimentin, cytokeratine, CD31, and CD34, findings consistent with a diagnosis of EAS (Fig 2). Thoracic and abdomino-pelvic CT imaging revealed lymphadenopathy in the axillary and iliac chains, prompting initiation of palliative chemotherapy. Given active local infection and poor wound status, definitive local radiotherapy was deferred by the multidisciplinary team owing to concerns about impaired wound healing and risk of worsening sepsis. She received systemic palliative chemotherapy consisting of weekly paclitaxel 80 mg/m^2^ administered intravenously on days 1, 8 and 15 of a 28-day cycle; 2 cycles (ie, 6 weekly doses) were completed. Baseline target lesions were measured on contrast enhanced CT and the sum of measurable target lesion diameters at baseline was 62 mm. Response assessment after 8 weeks showed an increased sum of 82 mm, representing a 32.2% increase, consistent with response evaluation criteria in solid tumors 1.1 progressive disease. In addition follow up CT imaging also revealed new regional lymph node involvement, and clinically there was no reduction in size or drainage of the lesions; therefore the multidisciplinary tumor board recommended amputation as a palliative measure for local control. Despite these interventions, the patient unfortunately succumbed to postoperative infectious complications.

## Discussion

STS is a rare and often fatal condition characterized by secondary angiosarcoma arising in the setting of chronic lymphedema of the extremity.^3^ Although STS is a well-recognized consequence of chronic lymphedema—most commonly after mastectomy and axillary dissection—reports in other etiologies of lymphedema (including obesity, paralysis, chronic infection and posttraumatic lymphedema) have been published. Overall there are only a few 100 cases reported in the literature, with the majority occurring in the upper limb after breast cancer surgery; lower limb cases are less common, and STS in the specific setting of RA-associated lymphedema is exceptionally rare with only isolated case reports and small series describing non-postmastectomy causes. Outcomes in published series remain poor: median survival is short (often measured in months to a few years), and prognosis is worse with regional or distant spread at diagnosis. Early biopsy of suspicious nodules, prompt imaging staging, and rapid multidisciplinary decision-making remain essential to improve chances of local control and survival.^4^

Angiosarcomas are uncommon malignant tumors originating from endothelial cells, classified into cutaneous, visceral, and soft tissue types, with EAS representing a particularly aggressive variant that primarily arises in deep soft tissues but can also originate in diverse sites such as the skin, adrenal glands, thyroid, and bone.^5^ EAS is rare and predominantly affects the head and neck region of elderly patients, characterized by a high propensity for lymph node metastasis and poor prognosis.^6^ EAS exhibits a wide morphological spectrum that poses diagnostic challenges on microscopy and appears to present differently in younger patients, often associated with lymphedema or vascular malformations.^7^ A noteworthy aspect of this case is the emergence of lymphedema linked to RA,^8^ which was subsequently complicated by angiosarcoma at a relatively young age—an outcome that is atypical. In fact, RA may induce profound degenerative alterations in the hip joint, potentially compounded by lower extremity lymphedema, thereby indicating the potential need for total hip replacement. Nevertheless, owing to the heightened risk of complications linked to this procedure, our patient declined to proceed with the intervention.^9^^,^^10^ In the years preceding her presentation, she was unable to ambulate normally and elected a predominantly bedridden lifestyle, which exacerbated her lower extremity lymphedema and intensified the associated venous stasis. Unfortunately, she also did not regularly utilize rehabilitation strategies, such as elastic bandages or compression stockings, to mitigate the progression of lymphedema, which ultimately led to worsening of her condition. In this clinical context, the risk of sarcoma development is heightened, as reports indicate that even in cases of localized lymphedema, the presence of cutaneous nodules should prompt suspicion for sarcoma. Although angiosarcoma can develop in lymphedema resulting from various etiologies, it has rarely been reported in association with RA-related lymphedema, where chronic inflammation may substantially elevate the risk and contribute to a poor prognosis.^11^^,^^12^ Our experience underscores the importance of maintaining heightened awareness for the possibility of angiosarcoma in this patient population when affected by lymphedema. It also highlights the critical role of rehabilitation strategies in controlling lymphedema, preventing its progression to a chronic state, and mitigating the risk of this fatal complication.

## Conclusion

This case illustrates the devastating progression of STS in the setting of untreated RA-related lymphedema, culminating in angiosarcoma, limb amputation, and fatal postoperative complications. It highlights the critical interplay between chronic inflammation, immobility, and lymphatic dysfunction in elevating sarcoma risk, particularly when rehabilitation interventions are overlooked. Clinicians managing RA patients with lower extremity edema must maintain a high index of suspicion for malignant transformation upon the emergence of cutaneous nodules and prioritize comprehensive lymphedema control through compression therapy, mobility enhancement, and timely surgical evaluation to avert such irreversible outcomes. Further research into the inflammatory mechanisms linking RA and angiosarcoma may inform targeted preventive strategies.

## Conflicts of interest

None disclosed.
